# Biographical Sketch of the Late Dr. James Gregory, of Edinburgh

**Published:** 1821-06-01

**Authors:** 


### *Biographical Notice of the late Professor James Gregory.*

Metam properamus ad unam.

Death, though repugnant to the hopes of the individual, and distressing to the feelings of friends and relatives, is yet one of the wisest and most beneficent appointments of an over-ruling Providence. The human mind cannot embrace the whole extent of misery which must inevitably ensue in this world, were man immortal, or even were the range of his existence much extended beyond the usual term. Strength would usurp power, and never let it go—talent would shoot up to an interminable height, and soon render all attempts or hopes of competition vain; of course, emulation would die, and one of the main springs of human action be withdrawn for ever. Wealth would follow the unequal division of power and reputation. There would be no intermediate shades between tyranny and slavery—riches and want—learning and ignorance. Nor would the possession of power, riches, or knowledge, continue *always* to confer happiness or pleasure on the favoured few. *Time* would indubitably take away, sooner or later, the enjoyment of all, and existence would become insipid, if not burthensome. It is no argument against this, that *finite* man is anxious to expand the little range of life which *he knows* is bounded to a span, and must soon terminate. It would be totally otherwise, had he no such fear; and after he had run the same rounds for a few centuries in succession.

As human reason itself, then, points out that Death is the great equalizer of good and evil, and furnishes the main stimulus to all human exertions, so is it equally unreasonable and unavailing to grieve when talent or learning is removed from this world, in the maturity of its growth. Every hour that it is continued beyond that period, it operates as a check on others, or robs them of their fair chance of acquiring similar distinction. This may appear a *cold-blooded* doctrine when viewed under the impression of local or personal feeling; but we are convinced that it is sound philosophy, and that its tendency is to reconcile us to the apparently hard lot of our nature, without checking the indulgence of any reasonable enjoyment of life.

The far-famed and long renowned University of Edinburgh has lost one of its brightest ornaments—perhaps one of its main pillars of support. What then?—Institutions must grow old as well as individuals, and ultimately share the common lot of humanity. Nor is this, according to our philosophy, to be considered as an evil, or deplored as a misfortune: far from it. The spirit of enquiry and the thirst of knowledge are principles too pervading at present, to permit the loss of any man, however transcendent his genius—or any alma mater, however extensive her influence, to check, for a moment, the current of instruction now flowing through such innumerable channels. Were the sun of talent to set (which we sincerely hope will never be the case) on the banks of the Forth, it would rise on the Clyde, the Liffey, the Thames, and the Seine. *Would rise*, did we say? It *has* risen. The monopoly of knowledge is dissolved; and as privileges are extended, competition must increase, so that nothing but actual and commanding talent can now sustain the reputation, power, or wealth, of a medical university. Let the senators of the intellectual city ponder on these things. Their wisest measures cannot prevent the competition around them, nor ensure the undiminished lustre of their school. But if they allow personal influence to supersede professional merit in their future elections, they may inscribe over the portals of their college—"TROJA FUIT."

But our admonitions to the living must not prevent our veneration of the dead. The desire of posthumous fame is a prominent feature, a predominant principle in the human mind. When fairly earned, then, during life, it should not be withheld, although censure cannot provoke the silent dust—

"Nor flattery soothe the dull cold ear of death."

Dr. James Gregory was descended from an ancient family in Aberdeenshire, his father having filled a professional chair in Aberdeen, and afterwards in Edinburgh. The subject of the present brief notice was born at Aberdeen in 1758, where he received the rudiments of his education. In 1766 he removed to the University of Edinburgh, and the following year was entered of Christ Church, Oxford, but soon returned to the northern metropolis. In 1773 he came to London to prosecute his professional studies, and became a pupil at St. George's Hospital. In 1774 he graduated at Edinburgh, the subject of his thesis being—"de morbis cœli mutatione medendis." During the year 1775, he was on the Continent, and visited Holland, France, and Italy. It is said that his letters from those countries to his friends at home, prove that he was feelingly alive to all the beauties of Nature, and the treasures of ancient art, there presented to his view. In 1776, at the early age of twenty-three, he was appointed to the professorship of the theory of physic, which he held with distinction for twelve years; and as a text-book for his lectures, published the first part of his Conspectus Medicinæ Theoreticæ, which was completed in 1782. This work acquired a great reputation—principally, we should think, on account of the felicity of classical language in which it is written. It is to be regretted, that in the successive editions the author never endeavoured to make it keep pace with the progress of medical science; hence its value is greatly diminished to the student. The death of Dr. Cullen, in 1790, raised Dr. Gregory to the chair of practical medicine, the celebrity of which he sustained for thirty-two years. "His lectures," says his biographer, "besides their intrinsic merit as the vehicles of sound medical learning, were characterized by a richness of illustration quite unprecedented." Dr. Gregory's memory was remarkably retentive, and he has been frequently heard to describe cases which had occurred in the earliest years of his practice with a freshness and particularity of detail which made them appear like the observations of the day. No case ever seems to have escaped his memory which could enforce some principle of pathology or practice; and he could draw, therefore, almost *ad libitum*, upon these stores, for striking illustrations of the doctrine he was inculcating. This was an attractive feature in the Lecturer, and always made a deep impression on the audience. How far Dr. Gregory's lectures aided the press in the diffusion of just notions in pathology, and bold measures in practice, we cannot form any probable estimate; —but doubtless they were powerful auxiliaries. The immense mass of medical information which he possessed, has perished with him; for the notes taken by his pupils are not only defective, but devoid of all his practical illustrations. We consider Dr. Gregory as highly culpable in not taking means to transmit as much as possible of his valuable knowledge to posterity through the medium of the press. Every man—and particularly every public man, in the confidence of his brethren and the plenitude of practice, should not merely put his talent out to advantage—but to the *best advantage*. This Dr. Gregory has not done; and it is greatly to be lamented that he had not dedicated those quartos of squabbling controversies to more dignified and professional pursuits!

We believe that for the last twenty years few physicians had more extensive practice; yet such was the liberality of Dr. Gregory's disposition, that his income was never what the celebrity of his name, and the number of his patients might give reason to expect. We believe, too, that "his generous heart was always open to afford that relief which wealth can bestow." We can easily conceive with his biographer, that "the loss of such a man will be felt as occasioning a blank almost irreparable in the academic celebrity of Edinburgh;" but we are not disposed to fear, with him, that the death of any individual can produce a similar blank in the "national distinction of the country."

We have studiously avoided the language of panegyric, which is feelingly employed by the biographer, though naturally and pardonably so in him, as dictated by friendship. Our feelings, of course, are under the guidance of cool contemplative philosophy, which views things in their general, rather than in their local, bearings, and loves to deduce moral lessons alike from evil and good, adversity and prosperity, death and life, vice and virtue—which, in fine, sees with the mind's eye—

> "Sermons in stones, books in the running brooks,
> "And good in every thing."
>
> SHAKESPEARE.

